# Electrophysiological and Anatomical Correlates of Spinal Cord Optical Coherence Tomography

**DOI:** 10.1371/journal.pone.0152539

**Published:** 2016-04-06

**Authors:** Mario E. Giardini, Antonio G. Zippo, Maurizio Valente, Nikola Krstajic, Gabriele E. M. Biella

**Affiliations:** 1 Department of Biomedical Engineering, University of Strathclyde, Wolfson Centre, 106 Rottenrow, Glasgow G4 0NW, United Kingdom; 2 Institute of Molecular Bioimaging and Physiology, National Research Council (CNR), Via Fratelli Cervi 93, 20090 Segrate (Milan), Italy; 3 CMOS Sensors Group, Integrated Micro & Nano Systems, School of Engineering, University of Edinburgh, The King's Buildings, Edinburgh EH9 3JL, United Kingdom; Simon Fraser University, CANADA

## Abstract

Despite the continuous improvement in medical imaging technology, visualizing the spinal cord poses severe problems due to structural or incidental causes, such as small access space and motion artifacts. In addition, positional guidance on the spinal cord is not commonly available during surgery, with the exception of neuronavigation techniques based on static pre-surgical data and of radiation-based methods, such as fluoroscopy. A fast, bedside, intraoperative real-time imaging, particularly necessary during the positioning of endoscopic probes or tools, is an unsolved issue. The objective of our work, performed on experimental rats, is to demonstrate potential intraoperative spinal cord imaging and probe guidance by optical coherence tomography (OCT). Concurrently, we aimed to demonstrate that the electromagnetic OCT irradiation exerted no particular effect at the neuronal and synaptic levels. OCT is a user-friendly, low-cost and endoscopy-compatible photonics-based imaging technique. In particular, by using a Fourier-domain OCT imager, operating at 850 nm wavelength and scanning transversally with respect to the spinal cord, we have been able to: 1) accurately image tissue structures in an animal model (muscle, spine bone, cerebro-spinal fluid, dura mater and spinal cord), and 2) identify the position of a recording microelectrode approaching and inserting into the cord tissue 3) check that the infrared radiation has no actual effect on the electrophysiological activity of spinal neurons. The technique, potentially extendable to full three-dimensional image reconstruction, shows prospective further application not only in endoscopic intraoperative analyses and for probe insertion guidance, but also in emergency and adverse situations (e.g. after trauma) for damage recognition, diagnosis and fast image-guided intervention.

## Introduction

As in all areas of the central nervous system, a key aspect in brain and spinal surgery is represented by the correct navigation of the surgical tools, including the positioning of exploring or electrophysiological needle probes in otherwise macroscopically homogeneous surgical fields [1]. For example, in spinal surgery, dural thickenings or adherences to the periosteum or trauma after-effects may hinder or block the advancement or the correct placement of probes. Avoidance of these interferences to probe insertion request direct, detailed and real-time discrimination of structures and microstructures of the cord and of its surroundings. This localization of anatomical reference points must be complemented by the visualization of probe trajectories during their positioning.

With the advent of spinal endoscopy (epiduroscopy), traditional image-guided techniques have been pushed to their limits, effectively highlighting substantial technical limitations [2]. The difficult access of the spinal canal, compounded with the intrinsic flexibility of the spine geometry, does not allow establishing a positional reference on preoperative data. Epiduroscopy therefore mandates the intensive use of continuous x-ray scanning, typically by C-arm fluoroscopy. Despite the growing safety of shielding strategies, the risk of radiation-induced damage to patients and medical personnel remains high [3–5]. Though radiation-less technologies, such as magnetic resonance imaging (MRI) guided interventions [6, 7] are actively being proposed on the spinal cord, they offer lower local resolution than required, and limited real-time capabilities. Indeed, successful computed tomography (CT) and MRI image guided navigation has been demonstrated in intraoperative applications, including cochlear implantation and facial fibrous dysplasia intervention [8, 9] yet this has not been done on the spinal cord. Furthermore, CT and MRI imaging exhibit prohibitive costs, complex infrastructure and surgical tools modified for compatibility with the imagers. A real-time, radiation-free method for routine fine probe navigation in spinal surgery intervention is indeed an urgent and, to the best of our knowledge, unmet requirement. Critically, this navigation method must not interfere with the functional (electrophysiological) properties of neurons.

Emerging imaging techniques, such as optical coherence tomography (OCT) [10] and photoacoustic imaging [11] appear to be particularly valuable in reducing the surgical invasiveness, whilst delivering real-time images of useful quality without the use of x-rays. In particular, with OCT it is indeed possible to visualize the morphology of tissue microstructures with accurate spatial discrimination (10 μm or better), with sufficient contrast for landmarking purposes [12, 13]. OCT lends itself to cost-effective implementation within small-diameter catheters and endoscopes, both in front-looking and in side-looking configurations [14]. Furthermore, no special adaptation of the surgical tools is required. OCT has been demonstrated to be clinically effective in other catheterized surgical contexts, such as in heart imaging for optimal stent coronary implantation [15, 16]. For these reasons, OCT appears as the ideal candidate for image-guided ambulatory and/or surgical epiduroscopic online guidance under continuous update of the intraoperative conditions.

Early studies on the application of OCT to intra-operative monitoring in neurosurgery demonstrated rapid feedback and ease of integration into surgical microscopes[17, 18]. In the past few years some crucial works on OCT imaging in the nervous system, including the spinal cord, has shown the feasibility of very detailed anatomical imaging, including the visualization of major and smaller diameter vessel arborizations [19, 20, 21]. Some authors have also shown the imaging of microelectrodes, although the images have been captured *a posteriori*, after electrode positioning in the nervous tissue, thus critically relying on *a priori* knowledge of the electrode or probe detection plane in order to obtain successful visualization, and with shadowing artifacts that obscure the anatomical landmarks in the very region around the electrode [22]. However, to the best of our knowledge, no work has been carried out showing *in vivo* OCT-guided probe advancement and insertion process while visualizing in real time the appropriate anatomical landmarks in the area of interest. Concurrently, no particular study has been carried out on the interference of OCT on the neuronal spontaneous and stimulated behavior during infrared probing.

In this preliminary experimental series carried out on experimental rats, after the surgical opening of the spinal canal, we have produced OCT-based spinal structure images highlighting distinctive landmarks such as muscles, bone and, before its removal, the intact dura mater and the liquor space. Additionally, under OCT control we have tracked the placement of thin electrophysiological microelectrodes throughout their insertion trajectory into the dorsal horn. Rats were chosen because of the fast model translatability of the spinal cord anatomical landmarks over the human anatomical environment, despite the challenging conditions of the spatially limited space. Furthermore, rat nervous tissue closely mimics the optical properties of human nervous tissue. Indeed, rats have traditionally been used as model system for tissue optics and optical probing [23]. Finally, in order to verify the absence of any interference of OCT electromagnetic emissions on the electrophysiological neuronal activity profiles, we performed recordings of the sensory neuronal spontaneous and light or high threshold peripheral stimulus related activities, in presence and in absence of OCT probing. The work has been carried out on surgically exposed vertebral canal, as preliminary proof of concept towards a potential final configuration in a closed surgery visualization system.

## Materials and Methods

### Ethical notes

All the animals have been treated along the Italian and European Laws on animal treatment in Scientific Research (Italian Bioethical Committee, Law Decree on the Treatment of Animals in Research, 27 Jan 1992, No. 116). The protocol adheres to the Guide for the Care and Use of Laboratory Animals (Institute for Laboratory Animal Research, National Research Council. Washington, DC: National Academy Press, 1996). The National Research Council, where the experiments have been performed, adheres to the International Committee on Laboratory Animal Science (ICLAS) on behalf of the United Nations Educational, Scientific and Cultural Organizations (UNESCO), the Council for International Organizations of Medical Sciences (CIOMS) and the International Union of Biological Sciences (IUBS). As such, no protocol-specific approval was specifically required for these specific experiments because it was included in the complete permission request to the Ministry of Health. The approval of the Ministry of Health has been classified as “Biella 1/2011” currently updated to “Biella 1/2014” into the files of the Ethical Committee of the University of Milan. The original communication of the Ministry of Health is referred to law decree of 27 January, 1992, n. 116, Actuative nr. 86/609/CEE. In the document S1 File is reported the “ARRIVE Guidelines Checklist”.

### Animal preparation and stereotaxis

Eight male albino rats (Sprague-Dawley, outbred strain, Charles River, Calco, LC, Italy, 275-300g) were employed in the experiments. Before the experimental sessions, the animals were maintained in 16/8 hours daylight-dark regimes, food and water ad libitum into the common animal facility of our departments (LITA, Laboratory of Advanced Interdisciplinary Technologies, LITA). We aimed to use the lowest number of animals in relation to the experimental needs (see the Subgroups of animals section below). The rats were hosted two in a cage in a non SPF sector and controlled once or twice a day by the responsible of the animal facility. Supervising visits from a veterinary doctor were carried out once a week. Some days were allowed to all the animals to rest after the animal delivery from the producing factory (Charles-River in Calco, LC, Lombardy). For the experiments, the rats were then taken singularly from their home cage and underwent preliminary barbiturate anesthesia (50mg/kg/1ml physiological solution) for the surgical experimental preparation. The trachea was cannulated to gain the connection to the anesthesia-ventilation device. The animals, mounted in a stereotactical frame, were then paralyzed by intravenous Gallamine thriethiodide (20 mg/kg/h) injected into the tail dorsal vein by a butterfly then left in place as continuous venous access. Concurrently, the rats were then connected to the respiratory device delivering (1stroke/s) an Isoflurane® (2.5% 0.8 to 1.5 l/min) and Oxygen (0.15–0.2 l/min) gaseous mixture. Curarization was maintained stable throughout the whole experiment by refracted Gallamine iv injections (0.1 ml of the original solution/h). During the experiment the anesthesia level was continuously monitored by EEG recordings. Before curarization, the anesthesia levels were maintained into ranges exhibiting absence of any corneal reflex or retraction reflex from the rats when checked with low intensity noxious mechanical stimuli applied on a posterior paw. The body temperature was maintained at 37.0 degrees Celsius with temperature controlled heat carbon-based resistive plates under the stereotactical bed. The heartbeat was continuously monitored by a heart rate recorder. All the experiments were carried out under strict ECG control. The experimental recordings have been prolonged and repeated as most as possible taking care of the levels of anesthesia, curarization and animal hydration to gather as most data possible from all the possible combination of the experimental protocols (electrophysiology and OCT independent and associated recordings).

### Spinal Cord opening surgery

With the animal mounted on the stereotaxic frame and placed under deep barbiturate anesthesia, the dorsal region was shaved and a surgical incision was made on the dorsal-lumbar midline. The long and short spinal muscle fascicles were progressively dissociated from their bone insertions until the complete vertebral exposure in the T12 –L4 spinal tract. After an accurate cleaning of the bone surfaces, the dorsal laminae and the spinous processes of the spine bone vault were carefully removed by a suitable rongeur in the vertebral tract L1 to L2, maintaining intact the *dura mater*. Clamping bars mounted on the side of the stereotaxic frame were used to block horizontally the spine by lateral hooking the vertebrae at T11-12 and L3-4, namely at the two ends of the exposed cord, under the transverse processes freed of the muscle tendons. In six animals, the meninges were delicately removed from the cord surface for comparative imaging with intact structure and for preparing the field for electrophysiological recordings.

### OCT and microelectrode recording sessions

Preliminary electrophysiological recording of the spinal neurons in the V lamina of the spinal cord were carried out by a single unit tungsten electrode (1 MΩ tip impedance at 1 KHz) to gather data on the spontaneous and evoked neuronal activity levels. The electrode was inserted into one half of the cord (ipsilateral to the posterior paw to be stimulated) in order to reach a vertical depth of 500 to 750 μm from the surface. The region between 500 and 750 μm has been chosen because of the highest density of Wide Dynamic Range neurons in the fifth spinal cord lamina, spanning just this width. WDR neurons are quite large ones, and potentially the most sizable of the dorsal horn of the cord and offer diffuse electrical field (in contrast with fusiform neurons that present polarized fields) easier to be detected and recorded in comparison to other neuronal types. In addition, WDR neurons express a wide range (hence their name) of frequencies in responding to both light and strong stimuli within an approximate proportionality coupling low frequency responses to light (mechanical or thermal) stimuli up to high frequency responses to noxious stimuli [24]. We could thus evaluate the effect of OCT scanning on the many response frequency bands expressed by the neurons. As for the interaction of the optical power with the neuronal responses the chosen depth was in the first fourth of the max depth reached by the irradiating beam, thus in the area presumably most strongly influenced by the scan. We had to introduce a 15° horizontal slant in order to avoid mechanical interference with the OCT probe placed orthogonally over the cord surface. These measures for reaching the V lamina were corrected by an elementary coordinate $\sqrt{6^{2}+0.5^{2}}$ = 6.25(along the varying vertical depth of the recorded neurons) = 6.25 to 6. 5]. In order to reach the correct recording depth, the electrodes were finely advanced by an electronically controlled microstepper M238 Linear Actuator and MS208E Motor Controller (Precision Instruments, Germany) with 1 micrometer step resolution, until full responses were observed to sensory test stimuli. Exploring stimuli were delivered to analyze the dynamic range of the recorded neurons either by repetitive touches with cotton tipped sticks or, interchangeably, by a thin paintbrush (light non-noxious stimuli) or by brief pinches by a graduated forceps over each single paw finger (noxious stimuli). The peripheral stimuli released to check the quality and quantitative features of the responses were in accordance with literature [25,26,27]. Measurements have been performed in absence and in presence of the OCT illumination beam. In different sessions, on the same animals, further sets of measurement have been done, alternating active/inactive settings. An interval has been always left between the measurement (as a rule some 5 mins between any measurement and the following). When the recorded neurons presented good responses in frequency to high and low threshold sensory mechanical stimuli, they were selected for the study. The recorded neuronal activity was attributed to Wide Dynamic Range (WDR) neurons, a class of neural cells extensively distributed in the deep dorsal laminae V and upper borders of VI and able to respond to a wide range of peripheral stimuli, [28]. The recordings were organized along fixed protocols. Briefly, for each animal, different experimental sessions have been performed with alternated spontaneous and stimulated activity recordings (low and high threshold stimuli, released at 1 Hz or 0.2 Hz frequency, respectively). The experimental stimuli were synchronized to the neuronal recordings through a sensible pressostatic probe applied on the paws connected to the recording device. The procedures have been repeated with active and inactive OCT probe.

The neuronal recordings were performed using a Neuralynx apparatus (Neuralynx Inc, USA) with 32 KHz sampling rate and high-low passbands at 300 Hz and 6 KHz or 0.1 to 6kHz respectively depending on the planned recording of lone spikes or with concurrent Local Field potentials (LFPs). After sampling and separation of neuronal signals by an unsupervised method for detecting and sorting spikes from multiunit recordings [29], we analyzed the spontaneous and evoked activities by representing them with Post-stimulus Time Histograms (PSTH). A sample of the recording activity in all conditions is available in the Supporting Information (S2 File).

The OCT images were recorded using a custom spectrometer-based Fourier-domain. The system is described in detail elsewhere [30, 31]. In brief, a single mode fiber coupled superluminescent diode with 850 nm central wavelength and 50 nm spectral bandwidth at half-maximum (SLED371-HP1, Superlum Diodes, Ireland) illuminates the tissue via a 3dB fiber coupler (FC850-40-50-APC, Thorlabs, NJ, USA), with a power on the sample on the order of 0.5 mW. A galvanometric scanner and an f-Θ microscope objective with working distance 25 mm and lateral resolution of 17 μm (LSM03-BB, Thorlabs, NJ, USA) are employed in the scanning head. The coupler divides the light between the probe and the reference arm. Backscattered spectra from the recombination of the reference and probe arms are detected by a custom transmissive spectrograph, containing a volume phase holographic grating (1200 l/mm, 830nm, Wasatch Photonics, USA) and a line CCD detector (AViiVA EM1, e2v, United Kingdom). The maximum A-scanning frequency is 30 kHz. Custom software, which controls the acquisition from CCD, performs the necessary signal processing and displays the image, was written in NI LabWindows 2009 (National Instruments, USA). The OCT sensitivity was measured to be 100 dB at 50 μs CCD integration time.

### Subgroups of animals

OCT recordings have been performed on two animals with the intact *dura mater*. In the other six animals, after the meninges removal, microelectrode electrophysiological recordings have been performed with active and inactive OCT (see above). In two cases the microelectrodes were finally retracted in order to assess the effects of their presence on OCT imaging

### OCT and Electrophysiology

The OCT probe was suspended over the stereotactical frame mounted on a mechanical micropositioner, in order to allow continuous acquisition from the surgical field. B-scans consisted of 512 to 2048 frames. B-scans were acquired perpendicularly to the spinal cord, in order to yield transversal images. In such configuration, the wave penetration depth is 0.8 to 1.2 mm, depending on the tissue. The axial resolution is 7 μm at the tissue surface, with a slight deterioration as the penetration depth increases.

The electrophysiological recordings have been repeated with and without OCT, in order to ascertain that the OCT infrared irradiation was not generating anomalous neuronal activities. OCT snapshots have been also taken during the guided microelectrode positioning into the cord. In particular, sets of 3 to 5 B-scans were taken in the direction of the spine axis, yielding an effective low-resolution C-scan. In our setup the frame rate was limited by the absence of a C-scan galvanometer. Yet, the low number of C-planes maintains the capability to track the electrode in 3 dimensions.

The OCT images have been analyzed using Gwyddion [32], an open-source microscopy-dedicated image processing software. In particular, the images have been equalized, and represented on a false-color scale as represented in the image color-bar.

### Spinal Cord extraction and Histology

**Fig 1:**
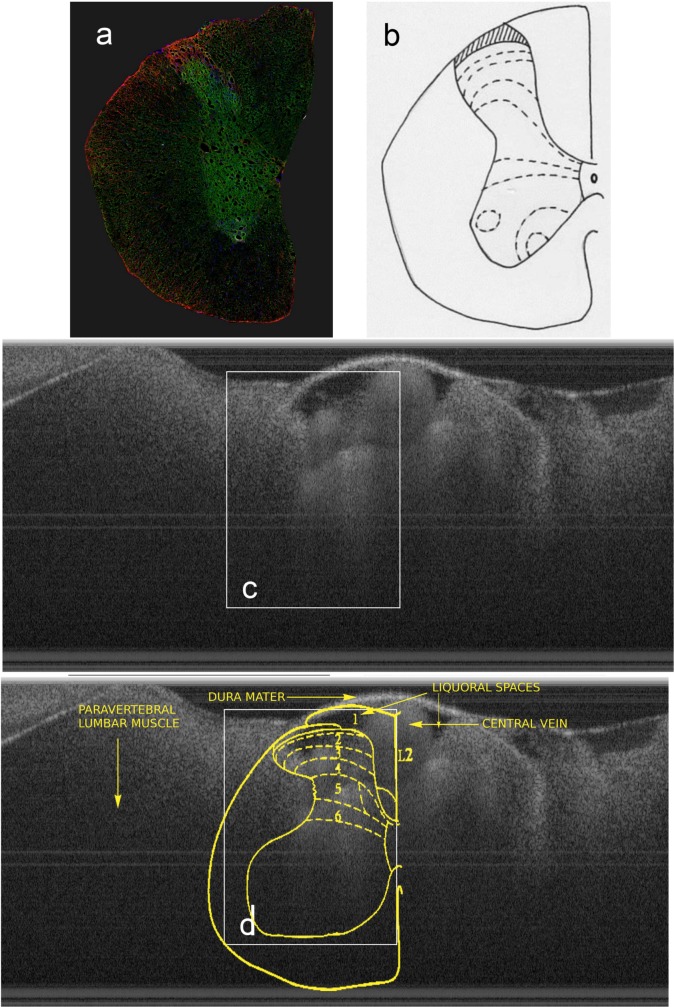
a) confocal image of a coronal hemisection of the spinal cord, with Glial Fibrillary Acidic Protein (GFAP, red), neurofilaments (green) distributed in the cytoplasm, and nuclei (blue); b) vignette to guide the eye, dotted lines delimiting the laminae; c) *in-vivo* cross sectional OCT image of the whole cord; d) laminar labelling superimposed over the OCT image in c). L2 means second lumbar myelomer and numbers from 1 to 6 refer to sensory laminae of the dorsal horn.

At the end of the experiments the animals have been sacrificed by deepening the anesthesia levels (Isoflurane up to 4l/min). A high dosage (150 mg/kg) Barbiturate (Nembutal) was injected. After the ECG control of cardiac death, the animals were rapidly removed from the stereotactic frame and placed over a grid for the intra-cardiac perfusion. Briefly, the animals were placed on their backs, taking care to not damage the exposed spinal cord. The thoracic plastron was excised and a small gauge needle with smoothed tip, connected to a tube from a peristaltic pump injecting perfusion liquids, was inserted into the left ventricle. The animals were perfused with 250 ml of heparinized physiological frozen solution and then perfused and fixed with a 4% solution of Paraformaldehyde in 1x Phosphate Buffered Saline (PBS). The spinal cord was delicately removed and placed in 1M PBS at pH 7.4. Slices of interest were obtained by a microtome. A confocal image (Leica, Germany) of a coronal hemisection of spinal cord is shown in Fig 1A with Glial Fibrillary Acidic Protein (GFAP, red) and neurofilaments (green) distributed in the cytoplasm, and the nuclei highlighted in blue.

## Results

All the data have been collected from germ-free animals not undergoing any pharmacologic treatment.

A representative cross-section of the spine, imaged with the intact dura at L2, is shown in Fig 1 compared to a confocal microscopy section of the same spine segment in Fig 1B and a vignette to guide the interpretation in Fig 1C. The OCT image can be clearly referenced to the histological image, substantially allowing the referencing of the OCT image to the anatomical landmarks.

**Fig 2:**
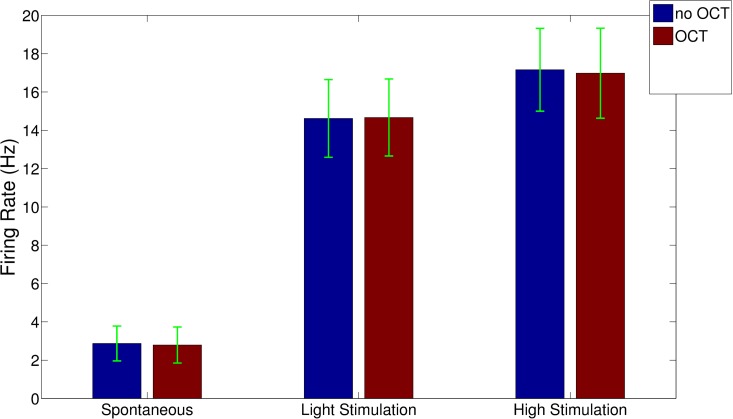
The mean spontaneous (left side) light (center) and high mechanical (right) threshold stimulus evoked mean neuronal activities. Blue bars: with inactive OCT probing. Brick-red bars: same as above in the presence of OCT scanning. Statistical tests (Wilcoxon non-parametric ranksum test) reported no significance between normal recordings and those performed in conjunction with OCT scanning [p = 0.564 (spontaneous), p = 0.890 (light stimulation), p = 0.711 (high stimulation)].

In Fig 2, the firing rates associated with the mean spontaneous and evoked activities of the responses to light tactile and noxious mechanical stimuli, in the presence and in absence of the OCT probe illumination beam are reported. No statistically significant significance is evident between the two conditions during spontaneous activity (p = 0.564, non-parametric Wilcoxon ranksum test), light (p = 0.890, ranksum test) and high threshold evoked activities (p = 0.711, ranksum test).

**Fig 3:**
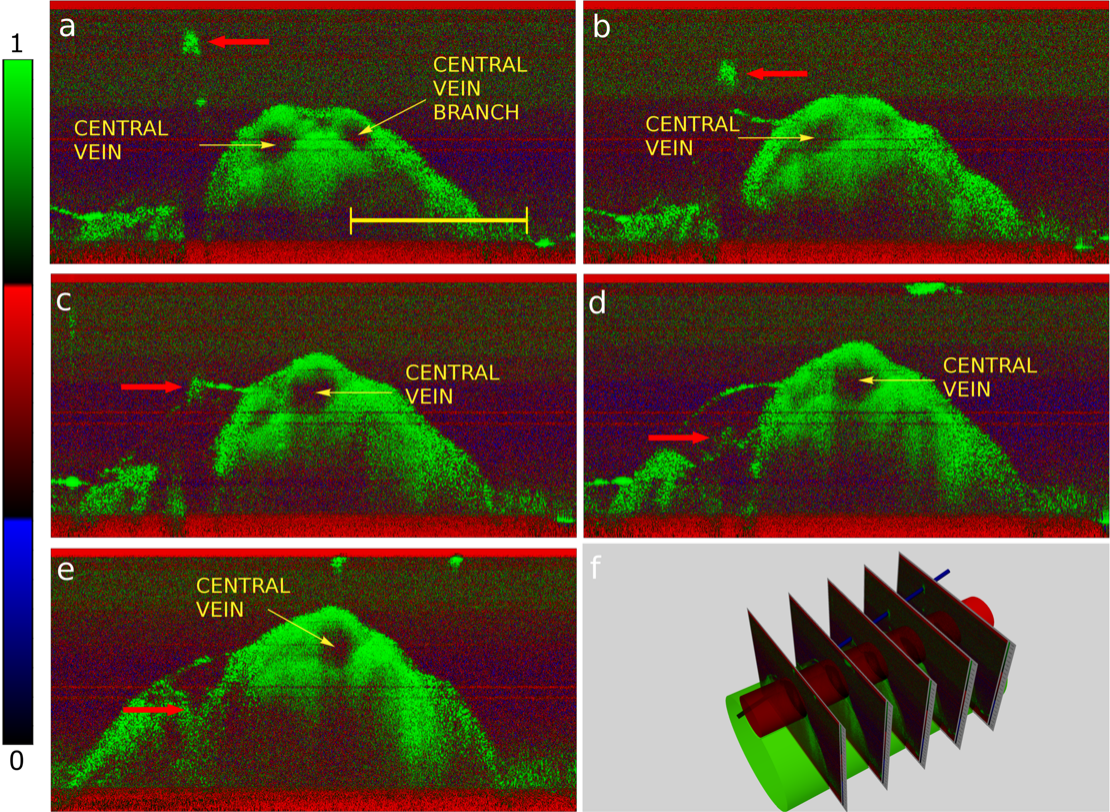
: a), b), c), d), e): OCT sequence of images showing the trace of a needle probe being inserted in the spinal cord (arrow), and f): mutual relation between the OCT images, cord, dorsal vein and needle (not to scale). In a), the OCT probe was aligned orthogonally to the tip of the microelectrode that progressively meet the axis of the central vein. The color bar indicates the color distribution over the normalized dynamic range. The scale bar is 3 mm long.

Fig 3 reports five OCT cross-sections acquired during electrode placement approaching the spine at 0.5 mm steps until the insertion into the cord (a to e). The electrode can be seen as a bright spot in each cross-section. As the electrode diameter is small (75–100μm), the shadowing effects are minimal, and the anatomical features are still visible below the electrode, but for a narrow dark stripe. The *dura mater* was left intact in the first stages of the electrode approach. The diagram (f) shows a coarse-grained cross-section tomographic reconstruction of the electrode trajectory. A brief animation of (f) is provided in the supporting information (see S1 Movie).

**Fig 4:**
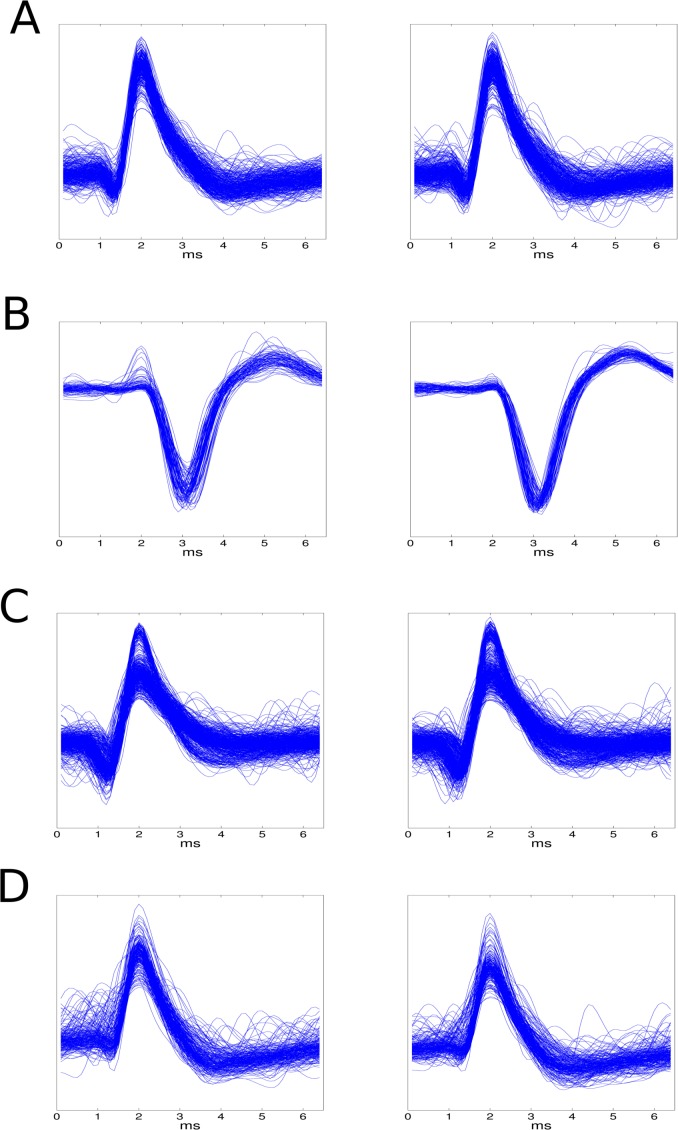
Spike shapes randomly chosen within the recorded data during the spontaneous, low and high threshold stimulus activations with active and inactive OCT probing.

We analyzed the spiking activity using the *Wave_clus* computational framework [29]. Spike waveforms were first extracted and subsequently reduced to their putative neurons. In this phase we explored whether the OCT electromagnetic emission could alter the recorded waveforms, provoking mismatches among the spike clustering. Among the 897 identified neurons, *Wave_clus* steadily coupled waveforms to neurons irrespectively to the on/off state of the OCT probe beam for 99.99% of neurons (893). By means of example, Fig 4 shows the spike waveforms of 4 putative neurons without (left column) and with OCT scanning (right column).

The absence of significant changes between the spike shapes recorded with active and inactive OCT scanning both in spontaneous and stimulus-evoked firing regimes, is suggestive of the absence of any functional interference of the infrared wavelengths over a wide range of the neuronal dynamic expression.

**Fig 5:**
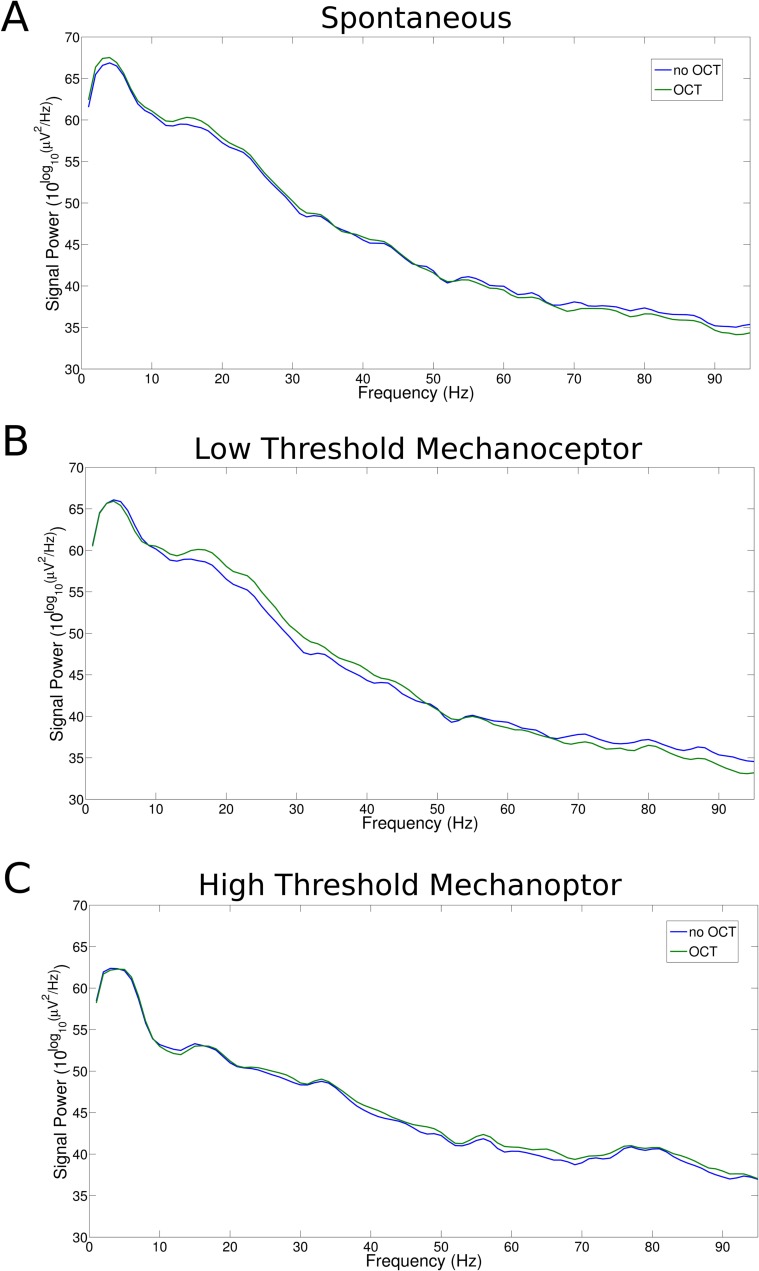
Local field potential spectral power in the same experimental conditions as above. No significant change was observable with active and inactive OCT probing.

To analyze further these aspects of electromagnetic interference by OCT on neuronal dynamics, a study has been carried out also on the Local Field Potentials (LFPs) of the recorded extracellular signal (Fig 5). Indeed, LFPs represent the integrated effect of many neural or neuro-glial communication episodes, the synaptic potentials being the most prominent. LFPs mainly manifest their activities in the 1–120 Hz frequency band. Again, even within these low frequency ranges, the OCT probing did not interfere with the normal activity. Indeed, the LFP power spectra in the three experimental conditions did not exhibit statistically significant differences. Analytically, the Kolmogorov-Smirnov p-values are p = 0.000 for spontaneous activity, p = 0.007, for light tactile activity and p = 0.001 under noxious stimulus.

All the analyses were collected from the whole set of experimental animals.

## Discussion

In this paper we show OCT (Optical Coherence Tomography) recordings from the lumbar segments of rat spinal cord in association with microelectrode electrophysiological recordings from the same tissue volumes. Though more complete results are needed to simulate closed-field conditions, this study has the aim to demonstrate in-vivo OCT scans in the spinal cord tissue simultaneous to the accurate OCT-basis tracing of thin exogenous probes, without reliance on *a priori* knowledge of the anatomy and/or the probe position. The exposed spinal cord after appropriate laminectomy can be correctly recognized (Fig 1C), as shown by the comparative examination of histological sections and confocal imaging of the same region (Fig 1A and 1B) from rats of comparable age and weight. Our measurements show that OCT is able to detect substructures of interest into the spinal cord and to differentiate between surrounding tissues. In this work, we purposely did not make use of the blood flow imaging features capabilities of OCT in order to refer the probes uniquely to spinal cord anatomical structures. Effectively, we have highlighted the structure of the dorsal horns yielding images compatible with post-mortem staining. Indeed, such structures are relevant to the probe intended location, and hence useable in navigation land marking for probe insertion. The anatomical features of interest, namely the meninges and the *dura mater* specifically, before it was removed, the central vein and the dorsal horns, are well observable from the OCT picture (Figs 1C and 3A–3E). In addition, using OCT we have successfully imaged an external probe approaching the spinal structures, referencing the trajectory to the anatomical landmarks in 3D (Fig 3F). Indeed, we have shown the possibility to use OCT in order to identify, image and follow electrode trajectories during the path towards and during the entry into the spinal cord. Our OCT device successfully detects the 80 um diameter microelectrodes used in these experiments (see Fig 3) and thus is potentially able to identify larger diameter probes. While imaging during the probe insertion, no *a priori* knowledge has been used in the identification of the insertion trajectory. The underlying anatomy has not been obscured by the probes, and it has been possible to use it to reference the probe position.

Recently, Kuo et al. were able to perform the epidural space identification in piglets with no direct access to the nervous structures [21]. Our study extends this to referencing and controlling a probe placement into the nervous tissue of the spinal cord in rats, where the optical properties and functional patterns of nervous tissue more closely resemble human tissue, and additionally to demonstrate that the OCT recording does not interfere with the electrical activity of spinal neurons (Fig 2). To this aim, the dynamic behavior of Wide Dynamic Range (WDR) neurons has been studied with and without the presence of the OCT scanning probe light beam. We chose the WDR neurons, a densely distributed neuron type particularly in the dorsal horn deep layers (the V lamina and the superior margin of the VI lamina) because their wide responsiveness to sensory peripheral inputs encompassing non-noxious and noxious stimuli (low and high threshold inputs). In detail, we restricted our search to the sole WDR neurons, because of the significant slant of the recording probes. Indeed, on the dorsal neurons, the electrode tip had to intercept almost orthogonally the electrical fields with a resulting reduction of the clarity. As a consequence, due to their bulky structure, the WDR neurons offered the highest cross section, maximizing the interception probability. Additionally, WDR offer optimal responsiveness to different stimulus intensities. Our measurements show that neither neuronal frequency band nor power spectrum has been affected by OCT (Figs 4 and 5) both at the neuronal and the LFP synaptic level in the two experimental conditions (inactive/active OCT probe). Spike shapes were also unmodified giving confidence that the delicate electrochemical and metabolic network sustaining the electrophysiological neuronal activity is unaffected by the infra-red probing, a clear benefit both in emergency interventional procedures and in the controlled environment of a surgery room. In addition, because spontaneous activity and evoked responses presented comparable spike composition with active and inactive OCT, this fact implies that even in different electrochemical conditions (as it happens in spontaneous and activated states) the OCT beam did not interfere with those processes [33]. Indeed, laser light has been shown to drive neurons in genetically engineered animals with cortical neurons sensitized to specific wavelengths [34]. In our much simple case, we are applying the infrared probe beam on onto a normal (not genetically engineered) structure, and our measurements confirm biological safety of the OCT probing even with respect to the delicate functional aspects of nervous tissue.

While our measurements have indeed been performed in open field surgery on a rat, the imaging penetration depth, in the millimeter range, is representative of the most frequent compressive or neoplastic masses in the spine, typically located epidural or subdural. In general, technology scaling to endoscopy and catheterization is well established in OCT [35], and demonstrating viability in open-field surgery remains the most critical step towards actual applicability. On the other side, the imaging needs further improvements to achieve more precise details if future spinal microsurgery applications are to be envisaged beyond these already available resources. Eventually, since the tissue optical features of human tissues are very close to those of rat tissues, typically used as model systems for tissue optics, we expected that comparable results could be envisaged. We note that, in our case the field of view exceeds the full spine while, in humans the relatively narrower field of view would need to be held into account.

### Conclusions

In conclusion, we have shown real time useful anatomical referencing OCT images obtained without *a priori* knowledge while following an electrode entry trajectory in the spinal cord. Additionally, we show that OCT probing does not interfere with the neural tissue function. Therefore, we hold that OCT shows the potential to become a viable referencing technology for safer and faster minimally-invasive spinal surgical guidance with intrinsic neurodynamic safety.

## Supporting Information

S1 File The ARRIVE Guidelines Checklist. (PDF)

S2 File The zip file contains six extracellular activities recorded in conjunction, or not, of the OCT probing. In the first case they contain “OCT” in the file name otherwise “noOCT”. In addition, files represent recordings in spontaneous activity condition (files have “spontaneous” prefix) or evoked condition (files have “evoked” prefix). Eventually, we selected two trials of both evoked conditions which files are labeled with the “trialX” suffix.
(DOCX)

S1 Movie 3D spatial representation of the cord and the recording electrode under different angular rotations. (DOCX)
