# Supplementary material for: Electrophysiological and Anatomical Correlates of Spinal Cord Optical Coherence Tomography
Source: PLoS One. 2016 Apr 6;11(4):e0152539. doi: 10.1371/journal.pone.0152539 (PMC4822845; doi:10.1371/journal.pone.0152539)
Supplement: S2 File — In the first case they contain “OCT” in the file name otherwise “noOCT”. In addition, files represent recordings in spontaneous activity condition (files have “spontaneous” prefix) or evoked condition (files have “evoked” prefix). Eventually, we selected two trials of both evoked conditions which files are labeled with the “trialX” suffix. (DOCX) [file pone.0152539.s002.docx]

**Supporting Information**

**Electrophysiological and anatomical correlates of spinal cord optical coherence tomography**

Mario E. Giardini^1#^, Antonio G. Zippo^2#^, Maurizio Valente^2^, Nikola Krstajic^3^ and Gabriele E.M. Biella^2*^

^1^Department of Biomedical Engineering, University of Strathclyde, Wolfson Centre, 106 Rottenrow, Glasgow G4 0NW, United Kingdom

^2^Institute of Molecular Bioimaging and Physiology, National Research Council (CNR), Via Fratelli Cervi 93, 20090 Segrate (Milan), Italy

^3^CMOS Sensors Group, Integrated Micro & Nano Systems, School of Engineering, University of Edinburgh, The King's Buildings, Edinburgh EH9 3JL, United Kingdom

mario.giardini@strath.ac.uk

antonio.zippo@gmail.com

mauriziovalenteuno@gmail.com

n.krstajic@ed.ac.uk

gembiella@gmail.com

*Corresponding Author:

Email: gembiella@gmail.com (preferred) (GEMB)

gabriele.biella@ibfm.cnr.it

# These two authors contributed equally to the work

**S2 File.** The zip file contains six extracellular activities recorded in conjunction, or not, of the OCT probing. In the first case they contain “OCT” in the file name otherwise “noOCT”. In addition, files represent recordings in spontaneous activity condition (files have “spontaneous” prefix) or evoked condition (files have “evoked” prefix). Eventually, we selected two trials of both evoked conditions which files are labeled with the “trialX” suffix.
