# Supplementary material for: Electrophysiological and Anatomical Correlates of Spinal Cord Optical Coherence Tomography
Source: PLoS One. 2016 Apr 6;11(4):e0152539. doi: 10.1371/journal.pone.0152539 (PMC4822845; doi:10.1371/journal.pone.0152539)
Supplement: S1 Movie — (DOCX) [file pone.0152539.s003.docx]

**Supporting Information**

**Electrophysiological and anatomical correlates of spinal cord optical coherence tomography**

Mario E. Giardini^1#^, Antonio G. Zippo^2#^, Maurizio Valente^2^, Nikola Krstajic^3^ and Gabriele E.M. Biella^2*^

^1^Department of Biomedical Engineering, University of Strathclyde, Wolfson Centre, 106 Rottenrow, Glasgow G4 0NW, United Kingdom

^2^Institute of Molecular Bioimaging and Physiology, National Research Council (CNR), Via Fratelli Cervi 93, 20090 Segrate (Milan), Italy

^3^CMOS Sensors Group, Integrated Micro & Nano Systems, School of Engineering, University of Edinburgh, The King's Buildings, Edinburgh EH9 3JL, United Kingdom

mario.giardini@strath.ac.uk

antonio.zippo@gmail.com

mauriziovalenteuno@gmail.com

n.krstajic@ed.ac.uk

gembiella@gmail.com

*Corresponding Author:

Email: gembiella@gmail.com (preferred) (GEMB)

gabriele.biella@ibfm.cnr.it

# These two authors contributed equally to the work

**S1 Movie.** 3D spatial representation of the cord and the recording electrode under different angular rotations.
